# Learning what matters: Synaptic plasticity with invariance to second-order input correlations

**DOI:** 10.1371/journal.pcbi.1011844

**Published:** 2024-02-12

**Authors:** Carlos Stein Naves de Brito, Wulfram Gerstner

**Affiliations:** 1 École Polytechnique Fédérale de Lausanne, EPFL, Lusanne, Switzerland; 2 Champalimaud Research, Champalimaud Centre for the Unknown, Lisbon, Portugal; Research Center Jülich, GERMANY

## Abstract

Cortical populations of neurons develop sparse representations adapted to the statistics of the environment. To learn efficient population codes, synaptic plasticity mechanisms must differentiate relevant latent features from spurious input correlations, which are omnipresent in cortical networks. Here, we develop a theory for sparse coding and synaptic plasticity that is invariant to second-order correlations in the input. Going beyond classical Hebbian learning, our learning objective explains the functional form of observed excitatory plasticity mechanisms, showing how Hebbian long-term depression (LTD) cancels the sensitivity to second-order correlations so that receptive fields become aligned with features hidden in higher-order statistics. Invariance to second-order correlations enhances the versatility of biologically realistic learning models, supporting optimal decoding from noisy inputs and sparse population coding from spatially correlated stimuli. In a spiking model with triplet spike-timing-dependent plasticity (STDP), we show that individual neurons can learn localized oriented receptive fields, circumventing the need for input preprocessing, such as whitening, or population-level lateral inhibition. The theory advances our understanding of local unsupervised learning in cortical circuits, offers new interpretations of the Bienenstock-Cooper-Munro and triplet STDP models, and assigns a specific functional role to synaptic LTD mechanisms in pyramidal neurons.

## Author summary

To understand how our brains carve out meaningful stimuli from a sea of sensory information, experimentalists often focus on individual neurons and their receptive fields; i.e., the input patterns that best stimulate the neurons. The receptive field of a neuron adapts to the statistics of the environment through plastic changes in the structure and strength of its incoming connections. Prevailing models of this adaptation, such as Sparse Coding and BCM theory, suggest that these connections are adjusted in a self-organized, unsupervised manner such that neurons can extract relatively rare, but complex patterns from the sensory input stream. Our theory extends these models, demonstrating that certain variants of the BCM model are capable of selectively ignoring pervasive second-order correlations and homing in on the pertinent, sparse signals in the stream of sensory stimuli. We have identified a carefully calibrated interaction between two aspects of synaptic plasticity: linear long-term depression (LTD) and nonlinear long-term potentiation (LTP). Such a balance enables a learning strategy that is sensitive to the complex statistical properties (higher-order correlations) of sensory inputs. In contrast to earlier approaches, so-called pre-whitening to remove second-order correlations is not necessary. Our findings provide not only a new framework for understanding the diverse roles of synaptic plasticity but also offer insights that may inform the development of artificial neural networks.

## Introduction

Cortical sensory areas contain rich representations of the external world, with individual neurons responding selectively to particular stimuli [1, 2]. These representations develop in early life and continue onward to adapt to the statistics of the environment [3]. While synaptic plasticity is thought to be central to cortical learning, it is still unknown how these biological processes can develop sensory representations. Normative models of sensory development, including sparse coding, independent component analysis, and Bienenstock-Cooper-Munro (BCM) plasticity [4–6], assume that cortical circuits self-organize to learn efficient sparse representations, aligning their receptive fields to sparse latent features hidden in the activity of the input population.

While synaptic plasticity models can be sensitive to sparse latent features, Hebbian mechanisms are also sensitive to input second-order correlations. Although mechanisms like retinal processing [7] and recurrent inhibition [8] can decorrelate neural activity, second-order correlations are widespread in cortical networks [9–11]. Under such conditions, input correlations (“second-order correlations”) can overshadow sparse latent features (“higher-order correlations”) so that learning will be dominated by input directions with the largest variance, akin to principal component analysis [12], developing selectivity for clusters of correlated inputs [13–15], but in general, failing to learn sparse representations [16, 17]. Several previous models of sparse sensory representations have side-stepped the issue by relying on assumptions of decorrelated inputs and identical firing rates, artificially removing input correlations through a preprocessing step referred to as ‘whitening’ [4, 18, 19].

Thus, it is still unknown how cortical learning mechanisms can learn from naturalistic input statistics, weeding out spurious input correlations while maintaining selectivity to higher-order correlations. Furthermore, the relation of plasticity rules derived from sparse coding models to experimental data remains often at a high level and cannot explain functional differences between plasticity mechanisms. In particular, the selective roles of homosynaptic LTD, on one side, and neuron-wide (heterosynaptic) depression mechanisms or homeostasis, on the other side, remain unclear [20–22].

Here we develop a theory of cortical unsupervised learning that selectively learns sparse latent features, taking into account the diverse statistics of presynaptic neurons. We demonstrate that invariance to (second-order) input correlations leads to biologically plausible plasticity mechanisms, requiring nonlinear Hebbian LTP and standard Hebbian LTD, linked with a homeostatic factor of meta-plasticity, including as special cases variations of the BCM [23, 24] and the triplet STDP models [25], classic models of excitatory plasticity. We show that this family of plasticity models optimizes an objective function, similar to that of sparse coding models [4, 26], but with the additional constraint of invariance to second-order correlations. Thus our objective function aims to selectively detect sparse features while ignoring potentially large second-order correlations in the synaptic input.

In simulations of increasing complexity, we demonstrate how invariance to second-order correlations enables biologically realistic models to learn efficient decoders and sparse population codes, developing synaptic weights that compensate for the noise of individual neurons, heterogeneity of firing rates across neurons, and correlated amplitude fluctuations of groups of neurons. Applied to sensory integration tasks, optimizing for sparsity translates to optimal integration of noisy inputs, weighing them according to their scale and reliability, leading to near-optimal linear decoders. In connected populations of neurons, the same plasticity rule leads to precisely tuned neurons even in cases where inputs have strong spatial correlations. Additionally, we adapted our theory to a spiking model of visual sensory development, with spiking neurons learning localized receptive fields from spatially correlated natural stimuli, even in the absence of decorrelating circuit mechanisms such as recurrent inhibition.

Learning with invariance to second-order correlations assigns a functional role to LTP, LTD, and homeostasis. In particular, linear Hebbian LTD is critical for invariance to second-order correlations, whereas alternative stability mechanisms, such as heterosynaptic plasticity [20, 27], do not confer correlation-invariance. Our theory provides a normative explanation for several distinct plasticity mechanisms in the brain. These results extend our understanding of how unsupervised learning with local Hebbian plasticity might be implemented in cortical circuits.

## Results

### Synaptic plasticity as sparse feature learning

We hypothesize that synaptic plasticity in single neurons implements an algorithm to learn features hidden in the input arriving in parallel at multiple synapses. In this view, the formation of receptive fields of sensory neurons during development is a manifestation of successful feature learning. We start by considering a simplified rate neuron *y*, with activation *y* = (**w**^*T*^**x**)_+_, receiving *N* inputs **x** = (*x*_1_, …, *x*_*N*_) through synaptic connections **w** = (*w*_1_, …, *w*_*N*_), where (.)_+_ denotes the rectified linear activation function, with activity *y* = **w**^*T*^**x** for **w**^*T*^**x** > 0 and *y* = 0 otherwise. We refer to the vector **w** of synaptic connections as the weight vector.

We assume that input features are characterised by sparse, non-Gaussian, statistics, as in sparse coding and independent component analysis (ICA) frameworks [4, 5]. Sparse statistics refer to long-tailed distributions, with a larger probability of atypical examples when compared to a Gaussian distribution with the same variance. For instance, the distribution for localized oriented filters in natural images can be modelled as a Laplace distribution, with longer tails than the distribution for a random filter [16, 28]. Features with infrequent all-or-none occurrences, as in a low-probability Bernoulli distribution, are also sparse. Since the linear mixture of sparse features is less sparse than the individual sources, we can use sparseness as an optimization principle to identify them [16]. As sparseness is determined by the shape of the distribution when normalized to unit variance, it is independent of second-order statistics. In contrast, the distribution of Gaussian components is entirely determined by its second-order properties. Under these assumptions, second-order correlations are uninformative about the latent features.

It is possible [19, 29, 30] to learn such features with local plasticity models provided the inputs have been decorrelated and normalized, i.e. whitened, by having been preprocessed to have an identity covariance matrix and unit firing rates. For such preprocessed inputs, it has been shown that a large class of sparsity maximization methods can retrieve the latent features [17]. Classically the sparseness of the output activity *y* is quantified by higher-order statistics, such as $\langle \frac{1}{4}y^{4}\rangle$, a measure related to kurtosis, where 〈.〉 denotes the expectation over the data samples {**x**}, or, more generally, by an objective function 〈*F*(*y*)〉, for some nonlinearity *F*(.) [29, 31]. An online plasticity rule (derived e.g. via stochastic gradient descent) converges to a solution that maximizes this objective, under the constraint of a normalized weight vector:

$$\begin{array}{l} \left(\text{1-a})\;\;\Delta w=\eta \;x\;f(y\right)\\ (\text{1-b})\;\;\;\;w\leftarrow \frac{w+\Delta w}{\left\|w+\Delta w\right\|} \end{array}\;\}\overset{converges}{\underset{\eta \to 0}{\Rightarrow }}\;w={\text{argmax}}_{w,|w|=1}\;\langle F\left(y\right)\rangle$$ (1)

where *η* is a learning rate and *f*(.) is the derivative of *F*(.). In general, this algorithm is robust to the specific shape of the nonlinearity *f*(.) [17, 31]. In particular, if $F\left(y\right)=\frac{1}{3}y^{3}$ then *f*(*y*) = *y*^2^, which relates to known experimental and theoretical results for activity-dependent models, as discussed below. The learning rule of Eq 1-a can be interpreted as a model of activity-dependent synaptic plasticity with a nonlinear Hebbian form of LTP [17]. Eq 1-b assures normalization of the weight vector and can be related to weight decay [32]. Normalization is a strict form of stabilization of the weight vector. A weaker form of stabilization can be achieved through dynamical mechanisms, such as heterosynaptic depression [27].

However, the simple sparsity objectives and related learning rules mentioned above do not learn the desired features if different input neurons have diverse firing rates or second-order correlations between them [17]. Instead of retrieving sparse features, they learn the input directions of the largest variance, as do PCA methods. Throughout the paper, we use the term ‘correlation’ without further specification to mean second-order correlation and mention higher-order correlation explicitly as such (e.g., ‘third-order correlation’).

### Theory of correlation-invariant learning

We aim for a synaptic plasticity rule capable of differentiating between relevant and irrelevant information, extracting low-amplitude sparse features even if synaptic inputs exhibit spurious second-order correlations of large amplitude. Here spurious refers to modulations with a Gaussian amplitude distribution whereas features are defined by a sparse non-Gaussian distribution.

As shown in Methods, an online update rule with LTP and LTD solves the *correlation-invariant* optimization problem $\langle \;F(\frac{y}{\sigma_{y}})\;\rangle =\langle \;{\left(\frac{y}{\sigma_{y}}\right)}^{3}\;\rangle$ in a rectified linear neuron *y* = (**w**^*T*^**x**)_+_:

$$\begin{array}{l} \left(\text{2-a})\;\;\Delta w=\eta \;(x\;y^{2}-h_{y}\;x\;y\right)\\ \left(\text{2-b})\;\;\Delta h_{y}=\eta_{h}\;(y^{2}-h_{y}\right) \end{array}\;\}\overset{converges}{\underset{\eta \to 0}{\Rightarrow }}\;w={\text{argmax}}_{w}\;\langle {\left(\frac{y}{\sigma_{y}}\right)}^{3}\rangle$$ (2)

Importantly, weight vectors are not constrained to norm one, but the output activity is normalized by its standard deviation, $\sigma_{y}=\sqrt{\langle \;y^{2}\;\rangle }$. We define *correlation-invariant* objectives as being invariant to the input correlations, and consequently invariant to linear transformations of the input such as rescaling or whitening, as demonstrated in Methods.

The plasticity model in Eq 2-(a,b) together with a rectified linear activation function is a variant of the BCM model with a dynamic threshold defined as *h*_*y*_ = 〈*y*^2^〉 [6, 33]. More generally, the property of correlation-invariance will hold for variants in which the neuron is linear or linear rectified and the LTP nonlinearity is a simple power-law, *x* *y*^*p*−1^, for all *p* > 2, p∈R, corresponding to the normalized objective $\langle {\left(\frac{y}{\sigma_{y}}\right)}^{p}\rangle$. In other words, the original BCM model [23] and later generalizations involving kurtosis optimization [6, 33], when implemented with a rectified linear activation function, are all part of a family of local learning rules with correlation-invariance that can be cast as optimization problems 〈*F*(*y*/*σ*_*y*_)〉. In contrast to learning rules derived from the objective in Eq 1 with normalized weight vector (as considered in ICA variants [16]), BCM variants do not normalize the weight vector but instead provide invariance to second-order correlations.

While invariant to correlations, this sparsity objective is still sensitive to the first-order statistics of the input, i.e. the input mean, which may dominate the learning objective. Following our assumption that the goal of excitatory plasticity is to learn higher-order statistics, we hypothesize that neurons subtract the input mean, and, accordingly, we normalize inputs to zero mean in all our simulations. Short-term depression [34] and spiking threshold adaptation [35] are candidate processes that might approximate input mean cancellation in cortical neurons.

Eq 2-a is a plasticity rule combining nonlinear Hebbian potentiation with linear Hebbian depression. Here, nonlinear (or linear) refers to the quadratic (respectively linear) dependence upon the activity *y* of the postsynaptic neuron. Importantly, the amplitude of the depression term is modulated by a metaplasticity function *h*_*y*_ that tracks the squared rate of the postsynaptic activity, 〈*y*^2^〉, estimated in Eq 2-b. We assume *η*_*h*_ ≫ *η* so that *h*_*y*_ converges more rapidly than the weights.

**Fig 1:**
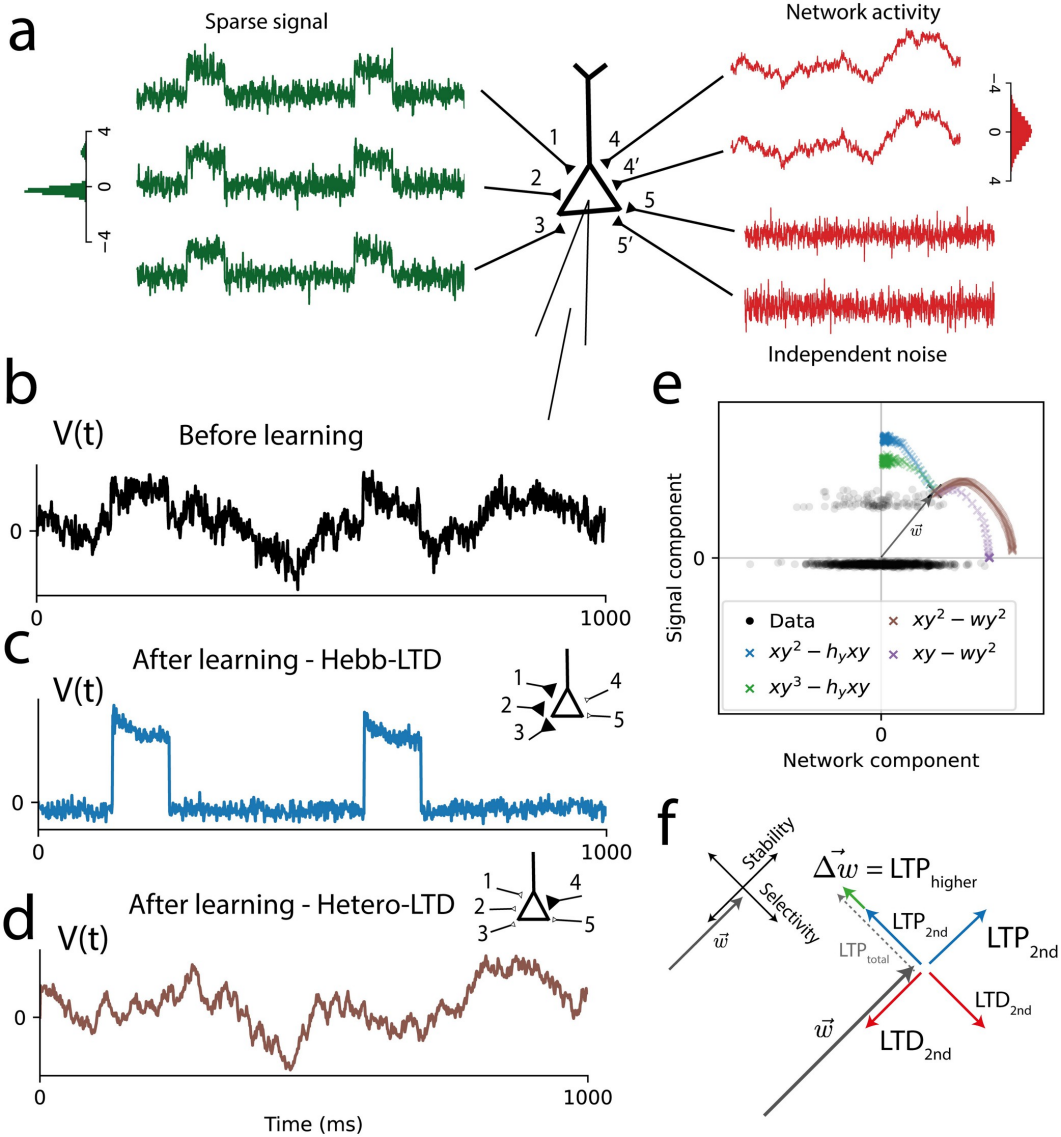
Learning sparse signals with correlation-invariance. A: Inputs belonging to three groups (20 inputs each): the sparse signal, with a non-Gaussian common component; the network activity, with a common Gaussian component, representing input from other brain areas; and independent background noise. Insets: histogram of amplitudes for sparse and network signals, with standard deviation *σ*_*Network*_ = 1.2 *σ*_*Sparse*_. B-D: Membrane potential as a function of time, before learning (black), and after learning, for the correlation-invariant model (BCM rule, blue) and the model with Oja-like heterosynaptic LTD (brown). Insets illustrate the synaptic strengths of each input group after learning. E: The learning dynamics of the weights (starting at the black X mark) projected to the Sparse Signal (y-axis) and Network (x-axis) components. A subset of data samples is shown in grey. The correlation-invariant rule (blue) converges to the direction of sparsest activity, as does the **x** *y*^3^ LTP variant (green). In contrast, the rule with heterosynaptic LTD (brown) and the original Oja rule (purple) converge to the direction of largest variance. This illustrates how the BCM model can perform Independent Component Analysis without a preprocessing step that decorrelates the inputs. F: To illustrate the mechanism behind correlation-invariance, we decompose the weights **w** into the stability and selectivity components. As the homeostatic mechanism balances LTP and LTD in the *stability component*, the LTD term cancels the exact amount of second-order dependency of the LTP term. Since in the orthogonal direction (*selectivity component*) the second-order components cancel as well, the net gradient Δ**w** (green) of the selectivity component depends only on the selectivity to higher-order statistics of the LTP term.

We illustrate the effect of correlation-invariance in a neuron receiving inputs from three sources, including a group of 20 inputs with a common sparse signal of unit amplitude, another group of 20 inputs with a common high-amplitude Gaussian signal, and the third group with small uncorrelated background activity (Fig 1A). The correlation-invariant learning rule learns the sparse signal despite its low amplitude, demonstrating selective sensitivity to higher-order correlations (Fig 1C and 1E). The above statements are equally valid for a kurtosis-style BCM model where Δ**w** = *η* (**x** *y*^3^ − *h*_*y*_ **x** *y*), with *h*_*y*_ = 〈*y*^3^〉 (Fig 1E). Thus there is a class of correlation-invariant rules as opposed to a single instantatiation of a rule (Methods). For comparison, we also simulate a similar plasticity model, but with a heterosynaptic LTD mechanism adapted from the Oja learning rule [12], Δ**w** = *η* (**x** *y*^2^ − **w** *y*^2^). Despite having a nonlinear LTP factor [17], this model learns the high-amplitude Gaussian component, as would a PCA model, and as does the original Oja rule, Δ**w** = *η* (**x** *y* − **w** *y*^2^) (Fig 1D and 1E). These simulation results illustrate that heterosynaptic LTD mechanisms provide stability, but not correlation-invariance.

### Linear LTD enables correlation-invariance

Numerous mechanisms have been proposed to account for the phenomenological properties of synaptic plasticity, but their specific properties and interactions are unclear [18, 27, 36, 37]. Previous work has shown that an effective nonlinear Hebbian LTP factor is a key mechanism for sparse feature learning, prevalent in many models [4, 5, 17, 38]. However, nonlinear Hebbian learning is not a sufficient mechanism, as illustrated by its failure in the presence of large second-order input correlations. Our theory of correlation-invariant learning enables us to extend these models to more general settings and assign distinct functional roles to LTP, LTD and homeostasis. Importantly, it is not sufficient to add any type of LTD or homeostasis to achieve a balance of LTP and LTD, but rather the above results indicate there is one preferred way of adding LTD that achieves a particularly smart balance because it normalizes second-order correlations instead of mean firing rates. And this specific form of LTD is linear in the pre- and postsynaptic firing rate. In other words, the LTD factor must be proportional to **x** *y* (and not to **x** *y*^2^ or **x**^2^*y*).

Let us recall the classic relationship between Hebbian learning and principal component analysis [12]. The PCA algorithm maximizes the variance in the input, with an objective function *F*(*y*) = 〈*y*^2^〉, for a linear neuron *y* = **w**^*T*^**x**, and can be implemented with a linear Hebbian learning rule, Δ**w** ∝ **x** *y*, with a positive proportionality constant. In contrast, in the class of correlation-invariant rules, the depression term is linear in pre- and postsynaptic activities, with a negative proportionality constant, −**x** *y*, which has the effect of removing the dependency on covariance from the learning rule, which we may call an “anti-PCA” effect. Therefore the online learning procedure will learn features independently of the input correlation profile.

To have complete correlation-invariance, the LTD mechanism must cancel the correct amount of second-order dependency. We can show (Methods, Eqs 20–26) that this is exactly what happens when the homeostatic factor *h*_*y*_ drives LTP and LTD to cancel each other in the direction of the weight vector. The component in the direction of the weight vector relates to the stability of the synaptic connections (i.e., the norm of the weight vector), and will be called ‘stability direction’ in the following. The orthogonal directions relate to feature selectivity, determining which feature has been learned. In Fig 1F, we give a geometric illustration for this mechanism in the 2-dimensional setting, decomposing the weights into the stability and selectivity components. The key insight is that changes in the stability component only scale the inputs, affecting only second-order statistics, while not altering normalized higher-order statistics. When the weight vector has approached its stable value, the LTD factor cancels the exact amount of the second-order dependency of the LTP factor in both components, leading to correlation-invariant learning. In contrast, heterosynaptic LTD is proportional to the weight vector **w** and does not act on the selectivity direction, leaving LTP selectivity dependent on second-order statistics. Importantly, any plasticity model that enforces normalization of the weight vector is unable to generically detect sparse features in the presence of second-order correlation because the norm of the weight vector needs to adjust itself to reflect the ratio of second-order and third-order correlations (or second-order and fourth-order correlations for the kurtosis model) and this ratio depends on properties of the signal that are not known a priori (Methods).

### Invariance to input amplitudes

**Fig 2:**
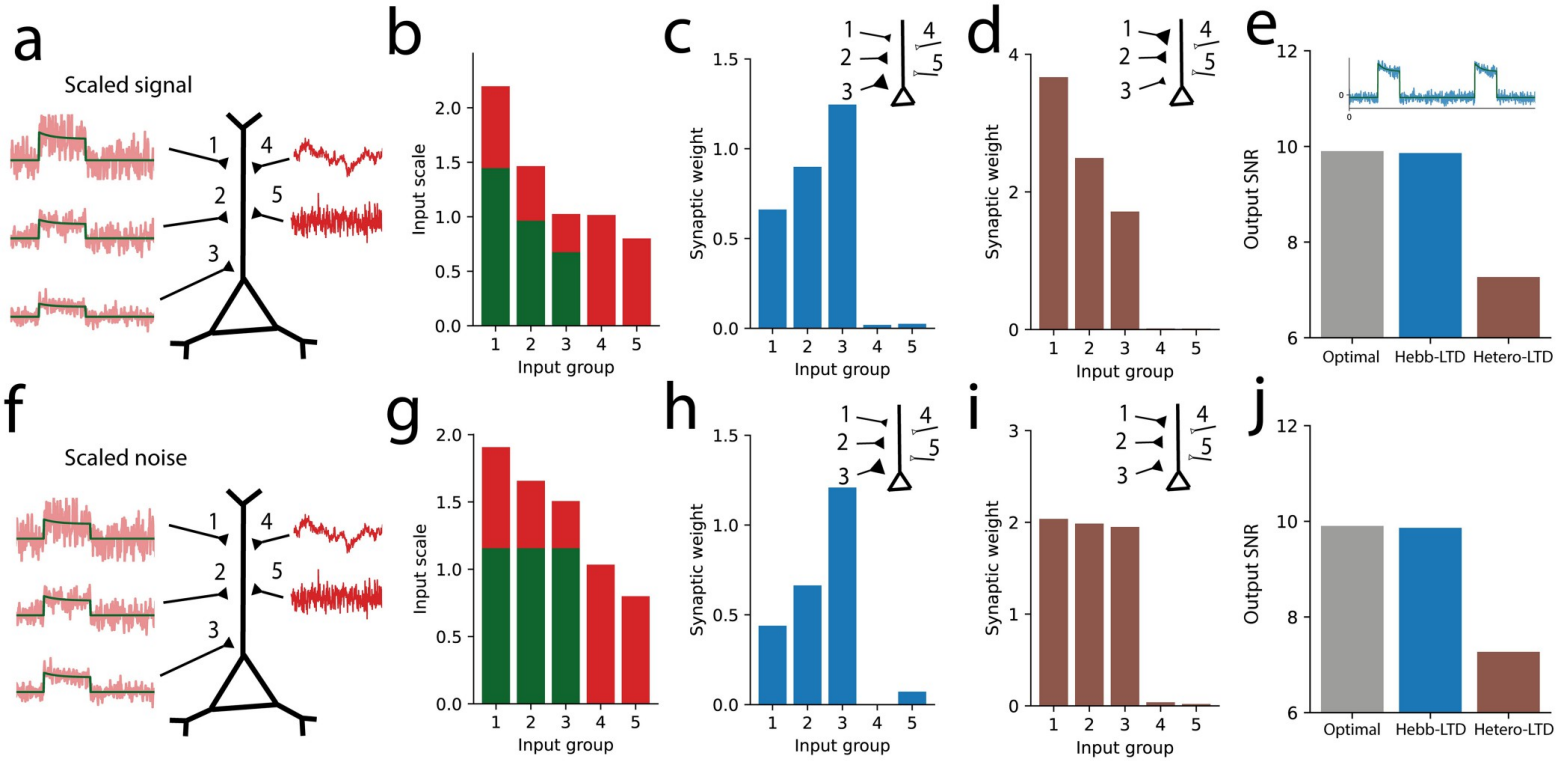
Optimal decoding under variable input scaling and noise. A: Sparse inputs with different amplitude levels but fixed signal-to-noise ratio (left, 1-3) and Gaussian-distributed inputs (right, 4 and 5). B: The sparse component group is divided into three subgroups with different standard deviations for their signal (green) and noise (red) levels, with the same signal-to-noise ratios. C: The correlation-invariant rule learns weights that compensate for the input scaling, with final weights inversely proportional to the input signal amplitude. D: The model with heterosynaptic LTD learns weights that are proportional to the input amplitudes. E: Inset: Output activity after learning with the correlation-invariant rule. Main graph: Signal-to-noise ratio of the output signal after learning with the correlation-invariant rule (Hebb-LTD, middle/blue), with heterosynaptic LTD (right/brown) and for the optimal linear decoder trained on the 5 groups of input channels (optimal, left). F: Sparse inputs with different noise levels, but fixed signal amplitude (left, 1-3). G: The sparse component group is divided into three subgroups with different noise levels (red), but the same signal amplitude (green). H: The correlation-invariant rule decodes the signal, learning synaptic weights proportional to the input signal-to-noise ratios. I: For comparison, the rule with heterosynaptic LTD learns weights proportionally to the input signal amplitude. J: As above, the correlation-invariant rule converges to a decoder almost as efficient as the optimal linear decoder.

Cortical neurons receive inputs from presynaptic neurons with complex firing statistics [39]. Many widely used plasticity models will fail to learn the expected features when different presynaptic neurons exhibit different scales of firing rate modulation since classic Hebbian learning is sensitive to the activity level of presynaptic neurons [12]. However, the correlation-invariant learning rule compensates for such differences. For example, let us assume that the sparse signal arrives at the different synapses with different amplitudes but always with the same signal-to-noise ratio (Fig 2A). In this case, each input has different second-order statistics (Fig 2B). After learning, the synaptic weights are inversely proportional to the signal amplitudes (Fig 2C), resulting in each input having the same contribution to the total input signal. We can quantify the efficiency of the learning rule by estimating the signal-to-noise ratio of the learned output signals, and compare it with that of an optimal linear decoder, trained with linear regression to output the sparse latent feature. We see that the correlation-invariant model achieves almost the optimal recovery of the latent signal (Fig 2E).

This invariance may be relevant for neurons with a large dendrite. For instance, the effect of input spikes on the somatic membrane potential is scaled down by dendritic attenuation, which varies with the distance from the synapse to the soma. It has been observed that synaptic strengths compensate for dendritic attenuation, and distal synapses have the same level of depolarization as proximal ones [40]. Note that we always simulate a point-neuron model. However, if we write the output as *y* = (∑_*j*_ *w*_*j*_*x*_*j*_)_+_, then we implicitly assume that the *x*_*j*_ represents the EPSP amplitude at the soma. In the presence of dendritic attenuation, the same synaptic current at the location of the synapse generates a smaller EPSP at the soma if the synapse is further away. Hence for the same signal-to-noise ratio in the input, the overall somatic amplitude would be smaller for a far-away synapse. When assuming dendritic attenuation, our model therefore predicts that a neuron with correlation-invariant plasticity will compensate for the attenuation, as it self-organizes the synaptic weights to compensate for linear disparities between synaptic inputs. Importantly, and in contrast with earlier work [41], this synaptic plasticity rule compensates for the difference in signal amplitude while staying sensitive to sparse features in the input, placing the compensation for dendritic attenuation within a normative framework.

### Optimal decoding from noisy inputs

When performing inference about a sensory variable, the brain integrates information from multiple unreliable sources, weighting them according to their reliability [42, 43]. To learn such an efficient decoder, neural circuits must be able to adapt incoming synapses according to the information conveyed by each input, searching for the most informative input combination. Conveniently, when decoding sparse latent variables, the direction with the highest signal-to-noise ratio will also be the direction with the sparsest distribution, which allows for classic ICA algorithms to also be applicable in the presence of noise [44]. Thus we can use our sparse learning objective to recover the most informative direction, using the correlation-invariant learning rule to learn an efficient decoder.

We simulated a neuron for which the inputs have variable signal-to-noise ratios (Fig 2F and 2G). The correlation-invariant learning rule develops weights proportional to the input signal-to-noise ratio, giving more importance to more informative inputs, leading to an output signal-to-noise level close to the optimal linear decoder (Fig 2H and 2J). Importantly, the plasticity rule does not simply select the one input synapse that has the highest signal-to-noise ratio but selects all input synapses that carry the signal, albeit with different importance weights. On the other hand, the learning rule with heterosynaptic LTD learns weights proportional to the input signal amplitude, with little sensitivity to input signal-to-noise levels (Fig 2I and 2J).

These results suggest that correlation-invariance could be a fundamental learning mechanism underlying near-optimal decoding from sensory information and multi-sensory integration, as seen in experiments [42, 43]. In comparison with related models based on maximal information transmission, such as independent component analysis [5], the correlation-invariant model requires minimal assumptions on the input distribution. A single plasticity rule learns an efficient decoder for different input scales, noise levels and sparse latent distributions.

### Learning sparse population codes from correlated inputs

While so far we have considered the learning properties of single neurons, sensory networks contain populations of neurons, with each neuron in the population representing different parts of the latent space, illustrated by the tuning curves of the population. Tuning curves for sensory signals are adapted to the statistic of input stimuli, and, in particular, are sharper for behaviorally relevant stimuli, providing a neural basis for efficient sensory discrimination [45–47]. As correlation-invariant plasticity leads to sparse and efficient responses, we demonstrate here its capability to learn sparse population codes for continuous stimuli encoded in a diverse, noisy input population.

**Fig 3:**
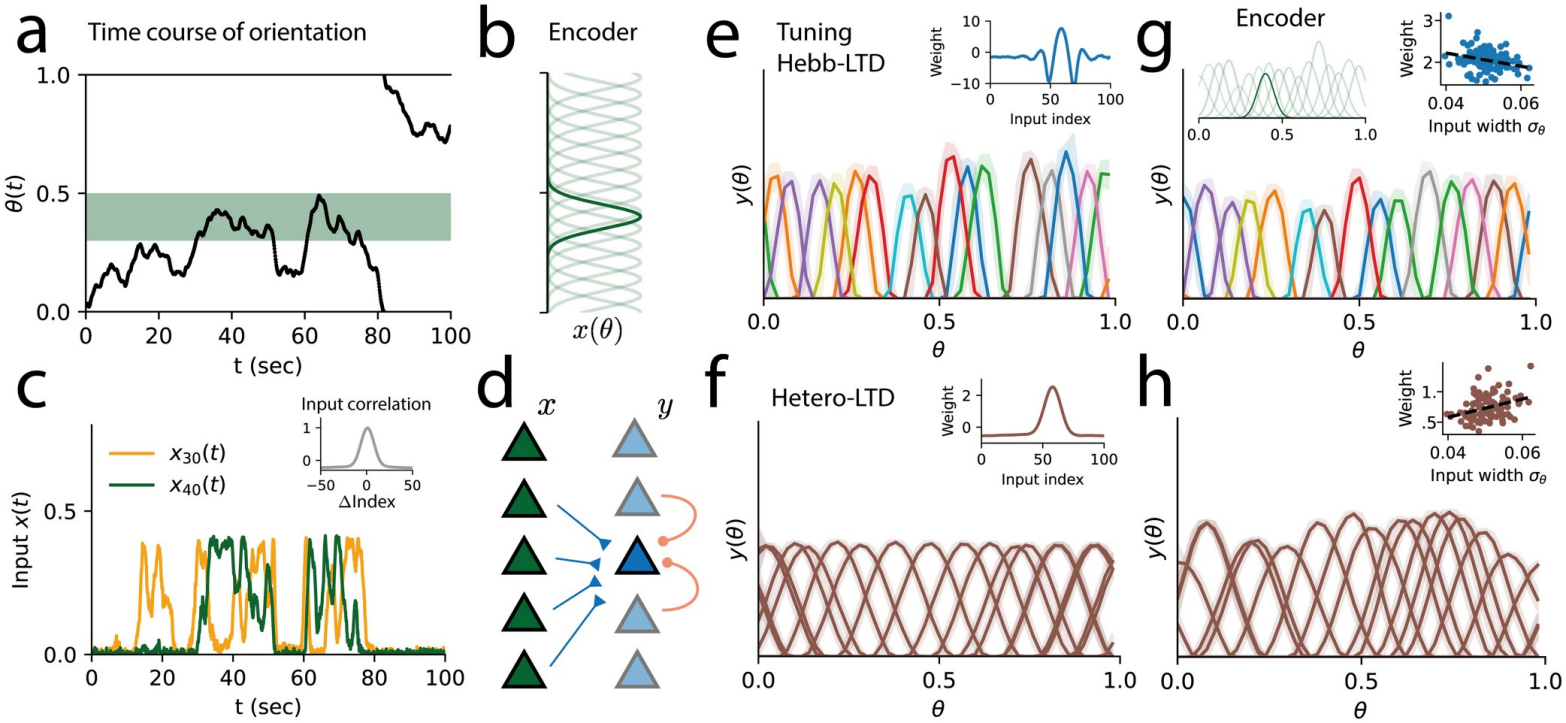
Correlation-invariant dictionary learning in a population coding network. A: A circular continuous latent variable follows a random walk with values between [0, 1]. B: The input population **x** encodes the latent variable with N = 100 Gaussian tuning curves **x**(*θ*(*t*)). C: The activity of two input neurons over time. Nearby inputs show positive correlations, following their overlap in tuning (inset, grey). D: Network diagram, with the input population (green) projecting synapses to a decoding population (blue). The synapses change according to the synaptic plasticity model and can take positive or negative values. Recurrent inhibition is included between all neurons (orange). E: The correlation-invariant model learns a dictionary of Mexican hat-like synaptic weights (inset, blue), inverting the input correlation profile, with tuning curves tiling the latent space with small overlaps between response profiles (coloured, variability in light shade). The population tuning curves are sharper (mean width at half maximum *σ*_*θ*_ = 0.07) than the tuning of input neurons (*σ*_*θ*_ = 0.11). F: With heterosynaptic LTD, neurons in the population learn synaptic weights (inset) following the input correlations, with wider tuning curves (*σ*_*θ*_ = 0.17) than those of input neurons. G: We simulate a new input population with heterogeneous tuning curves, with variation in width, amplitude and noise levels (inset, green). The correlation-invariant model learns again a sparse dictionary, optimizing for a sparse, low-noise representation. Tuning curves are sharper (*σ*_*θ*_ = 0.08) than the input tuning (*σ*_*θ*_ = 0.11), with higher selectivity for sharper input neurons (inset, correlation between input tuning width *σ*_*θ*_ and synaptic weight magnitudes: *ρ*_*σw*_ = −0.28). H: With heterosynaptic LTD, the population dictionary follows the input variance and correlation profile, learning wide tuning curves (*σ*_*θ*_ = 0.14), with higher weights for wider tuned input neurons (inset, *ρ*_*σw*_ = + 0.34).

We consider a line stimulus (or Gabor patch stimulus) that changes its orientation slowly over time. The stimulus is encoded by a noisy input population, with input tuning curves tiling the space of orientation angles (Fig 3A and 3B). As each input neuron is selective to only a part of the input space, they show sparse activity, with the overlap of the tuning curves generating positive input correlations between neighbouring neurons (Fig 3C).

We extend our single neuron model to a population of output neurons, with synapses from input to output population following the correlation-invariant plasticity rule of Eq 2 (Fig 3D). In cortical networks, recurrent inhibition is thought to decorrelate excitatory neurons, thereby allowing them to learn different features [26, 48, 49]. We thus include inhibitory recurrent connections between output neurons, which we consider a simplified effective description of the local excitatory-inhibitory network [50, 51]. Recurrent connections change with a covariance-based plasticity rule [48]. To avoid dynamic instabilities due to concurrent excitatory and inhibitory plasticity, we include multiplicative weight decay in both [20].

After learning, output neurons developed Mexican hat-like synaptic weight profiles, which have the effect of cancelling input correlations, leading to a population code tiling the space of line orientations with tuning curves sharper than those of inputs in the input layer (Fig 3E, mean tuning width *σ*_*θ*_ = 0.07; input tuning width *σ*_*θ*_ = 0.11). Following the same learning principles as in the single neuron case, the population code developed through learning can be interpreted as an efficient code with minimal redundancy. In comparison, the learning rule with heterosynaptic LTD learns wider tuning curves, which follow input directions of large variance, dominated by the input correlations (Fig 3F, *σ*_*θ*_ = 0.17).

Under more realistic conditions, sensory populations must decode information from neurons with diverse tuning properties. As we expect the correlation-invariant rule to be invariant to such input properties, we test the plasticity model in the presence of input heterogeneities. We simulated input tuning curves of variable widths, amplitudes and noise levels. As seen in Fig 3G, the correlation-invariant model learns a population code with similar properties (*σ*_*θ*_ = 0.08) as for homogeneous input tuning, with higher selectivity for more precise input neurons. On the other hand, a model without correlation-invariance learns wider tuning curves (*σ*_*θ*_ = 0.14), dependent on the input tuning profiles. In particular, neurons develop more selectivity for input neurons with wider tuning, disregarding their precision (Fig 3H).

We have shown that correlation-invariant plasticity leads to sharper tuning curves, which have been associated with adaptive neural responses [45–47]. Nevertheless, sharper tuning does not imply higher Fisher information, a metric used to estimate the efficiency of population code [52]. There is a complex relationship between the Fisher information and the sharpness of a population code, depending on latent dimensionality [53], the strength of lateral interactions [54], the number of neurons or decoding time [55] and the input tuning profile [56, 57]. In our model, the estimated Fisher information for the control network, with wider tuning curves, is higher than for the sharper code, learned by the correlation-invariant rule (*FI* = 38 ⋅ 10^3^ for control, *FI* = 36 ⋅ 10^3^ for correlation-invariant, for homogenous input tuning; *FI* = 38 ⋅ 10^3^ and *FI* = 33 ⋅ 10^3^, for heterogeneous inputs). This result illustrates that this model of cortical plasticity does not optimize Fisher information. While being an important metric for population codes, optimal Fisher information can have limited applicability when realistic constraints, such as wiring constraints and limitations in downstream readout populations, are considered [55].

### Spiking model of sensory development with correlated inputs

**Fig 4:**
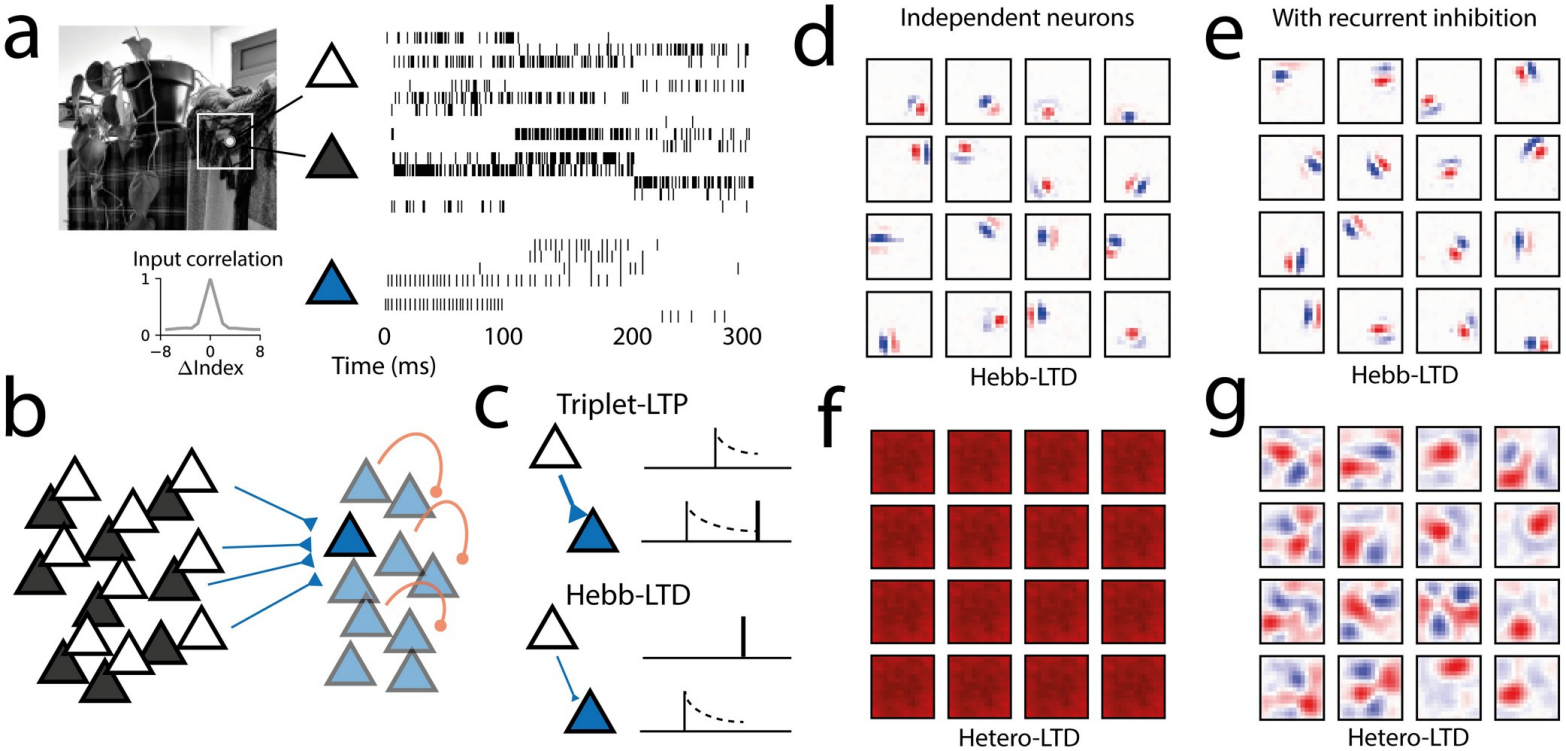
Correlation-invariant learning with triplet STDP facilitates receptive field development in a spiking network. A: Inputs were sampled from 16x16 patches of natural images (left), encoded as ON/OFF populations with Poisson spiking rates (right), representing visual input projections. The input has high pair-wise correlations for nearby pixels, and positive correlations over the whole patch (inset, grey) B: Spiking network model, with inputs projecting feed-forward excitatory weights to a population of 64 spiking neurons and recurrent inhibition. C: Excitatory weights are modified through triplet STDP, including the LTD mechanism linear on pre-post spiking correlations. D: Showing correlation-invariance, spiking neurons with triplet STDP learn localized receptive fields despite input correlations even in the absence of lateral inhibition. E: With recurrent inhibition included, neurons still learn similar receptive fields. F: Variation of the triplet STDP model with heterosynaptic LTD learns non-local input projections, due to sensitivity to input correlations. G: In the absence of correlation-invariance, lateral inhibition can promote somewhat more localized receptive fields, though still sensitive to the input correlation profile.

The relevance of a plasticity model comes from both the biological plausibility of the plasticity rule and from emerging functionality when embedded in plausible networks of spiking neurons. The correlation-invariant learning rule has a solid foundation in plasticity rules extracted from experimental data on cortical excitatory synapses. Cortical development is driven by voltage-dependent and spike-timing-dependent-plasticity (STDP), with synaptic changes depending on the relative timing of pre and post-synaptic spikes [58]. In particular, plasticity in excitatory synapses is modelled well by the voltage-based Clopath model [18] or the triplet STDP model [25], in which LTP depends on one pre- and two post-synaptic spikes, and LTD on single pre- and post-synaptic spikes (Fig 4C). The triple STDP model has been derived [25] from experimental data, in particular experiments with triplets of spikes and the frequency dependence of STDP [59, 60]. Considering a Poisson firing regime, and a homeostatic mechanism, the triplet model can, under rather general assumptions, be reduced to the rate model we have considered so far, Δ**w** = *η* (**x** *y*^2^ − *h*_*y*_ **x** *y*) [18, 25, 61]. From this relation, we might expect a spiking model of sensory development with triplet STDP to show correlation-invariance. Relative to rate models, spiking models are notoriously challenging to train, with added difficulty including spiking variability and spike-spike correlations [61]. Additionally, spiking constrains the input representation to be non-negative, changing how sensory information is processed.

We implemented a spiking network for sparse population coding, modelling V1 receptive field development from natural images (Fig 4A and 4B). It is a classic example where neurons develop selectivity to specific properties in their input, with the network creating a dictionary of localized orientation-selective features, which are the sparse features of natural images [4, 28]. Such models, however, do not differentiate between encoding higher-order and second-order correlations, and rely on inputs being preprocessed to be decorrelated and normalized [17]. Though the retinal pathway is known to partially decorrelate the visual stimuli, the input to cortical neurons still maintains some degree of correlation [9–11]. In the presence of spatial correlations, other models relied on recurrent inhibition, which diversifies the features learned by the network [19, 62]. In situations where single neurons or small networks learn the principal components (non-localized spatial Fourier filters) of the input images, features of sparse coding appeared only if recurrent inhibition was strong and the network was large enough [4, 19]. Motivated by the correlation-invariant theory, we wanted to test whether the STDP model can learn localized filters directly from natural stimuli containing spatial correlations, without invoking lateral inhibitory connections.

We considered an input dataset of natural image patches, encoded into ON and OFF spiking inputs, showing positive input correlations for neighbouring pixels (Fig 4A). Similarly to the rate model, we implement triplet STDP on input-to-output connections, output neurons modelled as leaky integrate-and-fire, and recurrent inhibition with inhibitory plasticity [48] (Fig 4B, see Methods).

To probe if learning was possible with single output neurons, we first ran the model without lateral inhibition. After learning, neurons developed localized receptive fields, composed of ON and OFF parts, similar to what is observed in V1 (Fig 4D), showing that the model can develop sparse features even without lateral inhibition. Even though each neuron has identical inputs, we see a diversity of receptive fields due to random initial conditions of the synaptic weights. When lateral inhibition was included, the model learned a similar dictionary of localized filters (Fig 4E). While lateral inhibition was not necessary in this setting for learning localized filters, it ensures the diversity of receptive fields [4, 17].

We compared our results with those of variation of the triplet STDP model, including a heterosynaptic LTD factor in place of the original Hebbian LTD factor, and found that in this case, all neurons learned a non-localized receptive field covering the whole patch, as is expected for a principal component of the input (Fig 4F). Only when lateral inhibition was included, did ON/OFF receptive fields appear, though not completely localized (Fig 4G). It demonstrates that without correlation-invariance, the spiking model is sensitive to input correlations, and requires lateral inhibition to enforce the tiling of the input space into sparse tuning curves. These results indicate that while correlation-invariance can be sufficient for learning sparse tuning curves, lateral inhibition can produce similar effects, with both mechanisms potentially at work in parallel in cortical circuits. In summary, by adding robustness to input noise, scaling and second-order correlations, the spike-based version of the correlation-invariant rule supports the spiking network to develop sensory representations with a diversity of localized receptive fields.

## Discussion

We have presented correlation-invariance as a critical property of cortical synaptic plasticity. Correlation-invariance is derived from the normative perspective of feature learning, in which cortical neurons develop responses to sparse latent features [4, 17, 26], while differentiating between high-order and second-order correlations. By discounting input correlations, we have shown how plastic cortical networks can solve efficient decoding tasks and learn sparse population codes with robustness and versatility.

Correlation-invariance stands in contrast to the original Hebbian learning perspective, grounded on learning by association [12, 63]. Instead, correlation-invariant models discount linear correlations, learning only higher-order correlations. The critical mechanism is a linear LTD factor, in agreement with models fitted to pairing experiments in excitatory synapses [18, 25, 59, 60], for which our results suggest a functional explanation. Our theory extends our previous understanding of Hebbian mechanisms and may aid the development of more complex representation learning models.

### A unifying theory for models of synaptic plasticity

Unifying theories have the potential to integrate diverse models, offering clarity on their relations as well as unique characteristics. They also highlight shared mechanisms essential to all models. For example, previous research emphasized the importance of a nonlinear LTP factor in synaptic plasticity, which enables sensitivity to higher-order statistics [17, 29]. Correlation-invariance theory introduces another crucial mechanism for sparse feature learning: a linear LTD factor that mitigates the effects of second-order input correlations. Consequently, our findings broaden the theory of synaptic plasticity exposing necessary mechanisms for the balance of LTP and LTD. Importantly, a linear LTD factor is consistent with the frequency dependence of STDP experiments as found by Sjostrom et al. [59] and described in the triplet STDP model [25].

Hebbian models such as BCM and Oja learning rules are decades old, and many studies have investigated their functional properties, concerning their stability, feature selectivity and receptive field development [24, 29, 33, 64]. In particular, BCM models have been motivated by their selectivity to higher-order statistics (enabled by its nonlinear LTP factor) and a meta-plastic stability mechanism. It has been observed that BCM variants can learn localized receptive fields when input images were preprocessed with a Difference of Gaussian filter, and thus are not wholly whitened, suggesting a lower sensitivity to second-order moments [33, 38]. In our derivations we have formalized these observations, revealing that some BCM models have in fact complete invariance to second-order statistics and that this is a key property to understand their distinct function.

In contrast, the original Oja’s rule [12] only learns second-order correlations (due to its linear LTP factor), while stability is achieved by synaptic depression using heterosynaptic weight scaling. Nevertheless, the functional difference between Oja’s heterosynaptic weight scaling and BCM’s anti-Hebbian depression factor has remained unclear. Our analysis shows that linear LTD allows for correlation-invariance in the feature direction, while Oja’s heterosynaptic weight scaling only acts on the stability component. Importantly, our results regarding the functional difference between weight scaling and linear LTD are to a large degree independent of the LTP model as long as the LTP model is nonlinear (e.g., *y*^2^ or *y*^3^) in the postsynaptic activity and linear in the presynaptic activity. This type of nonlinearity was predicted by the BCM model in 1982 [23] and confirmed by the analysis of experimental data via the triplet STDP model [25]. Therefore multiple theoretical plasticity models can now be unified in a theoretical framework and based on experimental data.

We have also uncovered an interesting relation between BCM, ICA and sparse coding, which are classic models of early sensory development. ICA and sparse coding start from similar normative assumptions, with inputs as mixtures of latent sparse features [65]. Our normalized objective function *F*(*y*/*σ*_*y*_) can be seen as an alternative to the standard formulation of sparse coding which usually defines a raw objective function *F*(*y*) where normalization of the weight vector is added as a further constraint [17]. Though the BCM model was first proposed as a stable version of Hebbian learning, we have shown that it links naturally to a normative formulation of sparse feature learning, with each of its elements seemingly designed for this task. We believe our theory provides a systematic basis for the analysis and development of Hebbian plasticity models.

Though our theory is based on a single-neuron objective, our network simulations demonstrate that correlation-invariant learning is compatible with learning network representations. It is essential to investigate how the theory of correlation-invariance might be integrated with related normative models for learning sparse, efficient representations [19, 49].

### Correlation-invariance in cortical neurons

The correlation-invariant learning rule has a precise correspondence to phenomenological models of spike-timing-dependent plasticity, including the triplet and voltage-dependent STDP models, which reduce to a quadratic postsynaptic factor for LTP and a linear postsynaptic factor for LTD [18, 25]. In particular, our theory suggests that pyramidal neurons should include synaptic LTD mechanisms linear in both pre and post-synaptic activities, in agreement with models of excitatory synapses [18, 25] fitted to data from pairing protocols [59, 60]. Since the experimental evidence for linear LTD factors is only indirect, inferred from the best-fitting models, it would be valuable to perform pairing experiments under Poisson firing times of pre and post-synaptic neurons to further investigate to what extent these properties hold [66].

Previous theoretical work has shown that the triplet STDP model generalizes the BCM model to the spatiotemporal domain [25], learning higher-order input spiking patterns, enabled by the nonlinear dependency on post-synaptic spikes [18, 61]. The models included a homeostatic factor *h*_*y*_ as a stabilizing mechanism, as was already mentioned in the original triplet STDP model [18, 25, 61]. However, going beyond a generic role in stabilization, in the current paper we have shown that the linearity of the LTD term is crucial in achieving feature selectivity in the presence of a potentially large amount of second-order correlations. The sliding of the factor *h*_*y*_ (equivalent to the ‘sliding threshold’ of the original BCM model [23]) has been interpreted in the past as metaplasticity or homeostasis. However, since traditional metaplasticity experiments have searched on slow time scales [20], it is unclear whether a rate detector exists that is fast enough to fulfil the function of the relatively fast sliding factor *h*_*y*_ [67]. In principle, stability may also be achieved through other mechanisms, such as heterosynaptic plasticity, though in this case, correlation-invariance will be partial and dependent on input statistics. In this case, there will be a compromise between learning higher-order and second-order statistics. Some sensitivity of plasticity rules to second-order statistics might be useful for other tasks, such as learning associative memories [27].

Our results are not in contradiction with the formation of Hebbian assemblies from correlations but rather give a refined view of how correlations drive assembly formation. Experimental tests of Hebbian assembly formation, also called memory engrams, have been performed in several brain regions [68–71]. Typically, a subset of neurons is switched on together during a training stimulus, which stops after some time. Such a switching process is very similar to the assumption in Fig 1A and induces a strong non-Gaussian distribution of firing rates and hence strong higher-order correlations. Thus our simulations are in line with existing experimental paradigms of assembly formation, even though Fig 1 focuses on a single postsynaptic neuron. In studies of recurrent networks of spiking neurons, several modelling papers have shown that variants of the triplet STDP rule, similar to the STDP rule or the BCM rate model in the present paper, give rise to the formation of Hebbian assemblies [18, 27, 72]. Again, the induction protocol used a switching process which generated not only second-order but also higher-order correlations. Importantly, our analytical insights predict that the formation of assemblies is dominantly driven by higher-order correlations and only weakly, or not at all, by second-order correlations. This insight is not in contradiction with earlier work, but suggests that statements such as ‘assembly formation is driven by correlation’ should be translated into the more precise statement ‘assembly formation is driven by higher-order correlations’. It also agrees with the functional role of higher-order correlations in theoretical STDP models [61, 73, 74].

Some findings on synaptic weight distribution provide evidence that cortical synapses self-organize with correlation-invariance. It has been observed that distal synapses are relatively up-regulated compared to proximal ones, and have in general somatic effects in the same order of magnitude as proximal connections [40]. Experiments on how synaptic profiles depend on input firing rates and correlations would be ideal to probe to which extent correlation-invariance might be at work in cortical circuits.

### Learning efficient population codes under diverse conditions

Experimental evidence indicates that primates can combine unreliable sensory information as would a near-optimal decoder [42, 43]. Normative population coding models approach this task by defining what each neuron represents about stimuli, for instance, the log-likelihood [75] or a probability distribution [76], from which a decoder can be designed. Such a design is difficult to learn with local rules, especially if inputs have unknown levels of reliability and correlations [77].

Instead, the correlation-invariant sparse objective operates at the algorithmic level, with minimal assumptions about how the input represents the latent variable. By assuming sparse latent variables, the objective becomes equivalent to maximizing the signal-to-noise ratio, and hence information transmission, enabling the development of population codes with sharp tuning and low noise. These properties do not imply, however, an optimization of the Fisher information for the population code [53–55]. How sparsity-based models relate to other normative population coding models is an important topic for further investigation.

### Search for biological learning algorithms

Representation learning is a difficult task and it is puzzling how the brain is capable of developing, maintaining and adapting a complex model of the external world. Only recently have artificial learning models been able to learn with very large, complex networks, but with methods that are not easily mapped to biological mechanisms [78, 79].

In the absence of supervising signals, unsupervised Hebbian plasticity provides the framework for learning a representation and may underlie how the cortex learns through local information [80–82]. Reinforcement learning is another central paradigm for understanding biological learning, believed to have a biological instantiation in neuromodulators and reward modulated plasticity. Indeed there is evidence in favour of the influence of reward-based learning on input representations and receptive fields in sensory cortices [83, 84]. It is an active field of research on how neuromodulators interact with Hebbian mechanisms [85–87]. It would be interesting to see how theories of sparse feature learning and correlation-invariance might be integrated with reinforcement learning objectives. Correlation-invariance extends the theory and function of Hebbian plasticity and might be an additional building block for models and theories of biological learning [88].

## Methods

### Linear invariance of the normalized objective function

We consider the unconstrained normalized projection pursuit objective (referred to as correlation-invariant objective), i.e. the output activity *y* is normalized and the total weight |**w**| norm is unspecified, of the form:

$$\begin{array}{c} w^{*}={\text{argmax}}_{w}\;\langle \;F(\frac{y}{\sigma_{y}})\;\rangle \end{array}$$ (3)

with $\sigma =\sqrt{\langle \;y^{2}\;\rangle }$ and *y* = *g*(**w**^*T*^**x**), for an output activation function *g*(.). We want to show the equivalence of this objective to a constrained unnormalized objective, for decorrelated inputs $\tilde{x}=Mx$, of the form

$$\begin{array}{c} {\tilde{w}}^{*}={\text{argmax}}_{\tilde{w},c\left(\tilde{w}\right)}\;\langle \;F(\tilde{y})\;\rangle \end{array}$$ (4)

for some constraint *c*, $\tilde{y}=g\left({\tilde{w}}^{T}\tilde{x}\right)$, with **M** being a transformation matrix for **x** that makes it decorrelated:

$$\begin{array}{c} \tilde{x}=Mx\Rightarrow \langle \;\tilde{x}{\tilde{x}}^{T}\;\rangle =I \end{array}$$ (5)

The transformation *M* is called *whitening* [89]. For instance, we can construct **M** = **RD**^−1/2^**R**^*T*^, where *D* is a diagonal matrix and 〈**xx**^*T*^〉 = **RDR**^*T*^ is the eigenvalue decomposition of the input correlation matrix.

We consider first a linear neuron, *y* = **w**^*T*^**x**. Using that $x=M^{-1}\tilde{x}$ and defining $\tilde{w}={\left(M^{-1}\right)}^{T}w$, we have

$$\begin{array}{c} \langle \;F(\frac{w^{T}x}{\sigma_{y}})\;\rangle =\langle \;F(\frac{w^{T}M^{-1}\tilde{x}}{\sqrt{\left\langle \;{\left(w^{T}M^{-1}\tilde{x}\right)}^{2}\;\right\rangle }})\;\rangle \end{array}$$ (6)

$$\begin{array}{c} =\langle \;F(\frac{{\tilde{w}}^{T}\tilde{x}}{\sqrt{\left\langle \;{\left({\tilde{w}}^{T}\tilde{x}\right)}^{2}\;\right\rangle }})\;\rangle \end{array}$$ (7)

$$\begin{array}{c} =\langle \;F(\frac{{\tilde{w}}^{T}}{|\tilde{w}|}\tilde{x})\;\rangle \end{array}$$ (8)

where we used Eq 5 to simplify the denominator: $\langle \;{\left({\tilde{w}}^{T}\tilde{x}\right)}^{2}\;\rangle =\langle \;{\tilde{w}}^{T}\tilde{x}{\tilde{x}}^{T}\tilde{w}\;\rangle ={\tilde{w}}^{T}\langle \;\tilde{x}{\tilde{x}}^{T}\;\rangle \tilde{w}={\tilde{w}}^{T}\tilde{w}={\left|\tilde{w}\right|}^{2}$. Thus the normalized objective function can be mapped to a standard objective function, with normalized weights and whitened inputs $\tilde{x}$,

$$\begin{array}{c} {\tilde{w}}^{*}={\text{argmax}}_{\tilde{w},\left|\tilde{w}\right|=1}\;\langle \;F({\tilde{w}}^{T}\tilde{x})\;\rangle \end{array}$$ (9)

with an optimum in the original input space given by $w^{*}=M^{T}{\tilde{w}}^{*}$.

Now considering a *general activation function*, *y* = *g*(**w**^*T*^**x**), we have an analogous derivation, however without the simplification of the denominator,

$$\begin{array}{cc} \langle \;F(\frac{g(w^{T}x)}{\sigma_{y}})\;\rangle = & \left\langle \;F(\frac{g({\tilde{w}}^{T}\tilde{x})}{\sqrt{\left\langle \;g{\left({\tilde{w}}^{T}\tilde{x}\right)}^{2}\;\right\rangle }})\;\right\rangle \end{array}$$ (10)

Thus the normalized objective function can be mapped to an unnormalized objective for whitened inputs $\tilde{x}$ and a constraint over the output standard deviation,

$$\begin{array}{c} {\tilde{w}}^{*}={\text{argmax}}_{\tilde{w},\sigma_{\tilde{y}}=1}\;\langle \;F(\tilde{y})\;\rangle \end{array}$$ (11)

and $\tilde{y}=g\left({\tilde{w}}^{T}\tilde{x}\right)$. Transforming the solution back into the original input space yields $w^{*}=M^{T}{\tilde{w}}^{*}$.

Analogously, given any linear transformation of the input, **x**′ = **Lx**, for an invertible matrix **L**, we may map the normalized projection pursuit to the whitened projection pursuit of Eq 11, with the optima given by $w^{\prime *}={\left(L^{-1}\right)}^{T}\;M^{T}\;{\tilde{w}}^{*}$. Hence, the normalized objective function of Eq 3 is invariant to linear transformations of the input.

### A correlation-invariant rule with arbitrary norm |w|

We consider *F*(*a*) = *a*^3^ with $a=\frac{y}{\sigma_{y}}$ and search for the optimal weight vector

$$\begin{array}{c} w^{*}={\text{argmax}}_{w}\;\langle \;{\left(\frac{y}{\sigma_{y}}\right)}^{3}\;\rangle \end{array}$$ (12)

assuming that the neuron has a *rectified linear activation function* *y* = (**w**^*T*^**x**)_+_ and where $\sigma_{y}=\sqrt{\langle y^{2}\rangle }$.

The normalized skewness contrast function has been considered in a variant of the BCM model (for a sigmoid activation function instead of a linear rectifier), and the derivations below follow similar steps to derive an online learning rule from it [23, 33]. Proceeding with gradient ascent on **w**, we have

$$\begin{array}{c} \frac{\partial \langle \;F\;\rangle }{\partial w}=\frac{\partial }{\partial w}\langle \;{\left(\frac{y}{\sigma_{y}}\right)}^{3}\;\rangle \end{array}$$ (13)

$$\begin{array}{c} =3\langle \;{\left(\frac{y}{\sigma_{y}}\right)}^{2}(\sigma_{y}^{-1}\frac{\partial y}{\partial w}+y\frac{\partial \sigma_{y}^{-1}}{\partial w})\;\rangle \end{array}$$ (14)

$$\begin{array}{c} =\frac{3}{\sigma_{y}^{2}}\langle \;y^{2}(\frac{1}{\sigma_{y}}\frac{\partial y}{\partial w}-\frac{y}{\sigma_{y}^{2}}\frac{\partial \sigma_{y}}{\partial w})\;\rangle \end{array}$$ (15)

We now use that the neuron has a rectified linear activation function so that $\frac{\partial y}{\partial w}=x_{+}$ and $\frac{\partial \sigma_{y}}{\partial w}=\frac{\partial \sqrt{\langle y^{2}\rangle }}{\partial w}=\langle yx_{+}\rangle /\sigma_{y}$, where we define **x**_+_ = **x** **I**_*y*>0_ as the input for samples in which *y* ≥ 0. Since the output of the neuron is always non-negative, we have *y* ≥ 0 for all **x** so that we have **x**_+_*y* = **x** *y* and **x**_+_*y*^2^ = **x** *y*^2^. This yields

$$\begin{array}{c} \frac{\partial \langle \;F\;\rangle }{\partial w}=\frac{3}{\sigma_{y}^{2}}\langle \;(\frac{x_{+}y^{2}}{\sigma_{y}}-\frac{y^{3}}{\sigma_{y}^{3}}\left\langle x_{+}y\right\rangle )\;\rangle \end{array}$$ (16)

$$\begin{array}{c} =\frac{3}{\sigma_{y}^{3}}(\left\langle xy^{2}\right\rangle -\frac{\left\langle y^{3}\right\rangle }{\left\langle y^{2}\right\rangle }\left\langle xy\right\rangle ) \end{array}$$ (17)

To derive an online learning rule, we consider a separation of time scales and assume that the estimation of *σ*_*y*_ and $\frac{\langle y^{3}\rangle }{\langle y^{2}\rangle }$ is performed at a faster time scale than the other factors, which allows us to consider them as constants. We derive a stochastic gradient ascent learning dynamics by removing the estimation over the whole dataset,

$$\begin{array}{c} \Delta w\propto x\;y^{2}-h_{y}\;x\;y \end{array}$$ (18)

We refer to the specific choice $h_{y}^{*}=\frac{\langle y^{3}\rangle }{\langle y^{2}\rangle }$ as the *balancing homeostatic factor*. We claim that the balancing homeostatic factor leaves the learning rule at an *indifferent stability* in the direction of the weights, leaving the norm fluctuating freely. We can check this property by showing that the gradient in the direction of the synaptic connections is zero when averaged over the full dataset,

$$\begin{array}{c} \left\langle w^{T}\Delta w\right\rangle \propto \left\langle y^{3}\right\rangle -h_{y}^{*}\left\langle y^{2}\right\rangle =0 \end{array}$$ (19)

It is a consequence of using an objective function that is invariant to the norm of the weight vector.

### A family of correlation-invariant learning rules with stable weights

While the top-down derivation of the correlation-invariant learning rule leads to a specific balancing homeostatic factor $h_{y}^{*}=\frac{\langle y^{3}\rangle }{\langle y^{2}\rangle }$, it is not a stable learning rule, as the norm of the weight vector will fluctuate freely. Instead, we can consider factors that are stable, such as *h*_*y*_ = 〈*y*^2^〉. In fact, any supralinear factor *h*_*y*_ = 〈*y*^*r*^〉, with *r* > 1, will lead to stable dynamics [6, 23]. We claim that the family of stable plasticity rules with these alternative homeostatic factors will, after convergence, optimize the same objective function as the learning rule derived in the previous paragraph. To demonstrate this, we calculate the homeostatic factor once the norm has converged to a stable value. Under the assumption that the gradient in the direction of the weights **w** is zero, we find

$$\begin{array}{c} \left\langle w^{T}\Delta w\right\rangle \propto \left\langle y^{3}\right\rangle -h_{y}\left\langle y^{2}\right\rangle =0\Rightarrow h_{y}=\left\langle y^{3}\right\rangle /\left\langle y^{2}\right\rangle =h_{y}^{*}\left(y\right) \end{array}$$ (20)

which implies that when the weight norm has approached a stable value during the learning process, the stabilizing homeostatic factor *h*_*y*_ will have the same value as the balancing homeostatic factor $h_{y}^{*}$ for the same weights, and consequently will have the same correlation-invariant properties.

Critically for the invariance properties in Eqs 20 and 19 to hold, we used that (i) a power-law sparsity function *F*(*a*) = *a*^*p*^ can be written as *F*(*a*) = *a* *F*′(*a*)/*p* for all *p* > 2 with p∈R and (ii) a linear or rectified linear transfer function can be written as *g*(**w**^*T*^**x**) = (**w**^*T*^**x**) *g*′(**w**^*T*^**x**), where *F*′ and *g*′ are the derivatives of *F* and *g*, respectively. We note that for a rectified linear transfer function *g*′ is either zero or one. Together these properties yield a rewrite

$$\begin{array}{c} F\left(g\left(w^{T}x\right)\right)=\left(1/p\right)\;F^{\prime }\left(g\left(w^{T}x\right)\right)\;g^{\prime }\left(w^{T}x\right)\;w^{T}x \end{array}$$ (21)

In contrast, a sigmoidal activation function *y* = *σ*(**w**^*T*^**x**), as in the original BCM model [23], or a different LTP nonlinearity, e.g. **x** (*y* − 1)_+_, will generally not satisfy these properties and therefore do not generically lead to complete correlation-invariance.

In summary, we have shown that the family of correlation-invariant learning rules of the form:

$$\begin{array}{c} \Delta w\propto x\;y^{p-1}-h_{y}\;x\;y \end{array}$$ (22)

with *y* = **w**^*T*^**x** or *y* = (**w**^*T*^**x**)_+_, *h*_*y*_ = 〈*y*^*r*^〉, for any *p* > 2 and *r* > *p* − 2, where p,r∈R, will converge to $w^{*}=M^{T}{\tilde{w}}^{*}$, where ${\tilde{w}}^{*}$ is a local optimum of the constrained objective for whitened inputs $\tilde{x}=Mx$, with $\langle \;\tilde{x}{\tilde{x}}^{T}\;\rangle =I$:

$$\begin{array}{c} {\tilde{w}}^{*}={\text{argmax}}_{\tilde{w},\sigma_{\tilde{y}}=1}\;\langle \;{\tilde{y}}^{p}\;\rangle \end{array}$$ (23)

*Therefore, any plasticity rule of the form* (22) *in a linear or rectified linear neuron model will be insensitive to second-order correlations in the input*.

We can also calculate analytically the norm the weights will have during the learning process. For *h*_*y*_ = 〈*y*^2^〉, we have

$$\begin{array}{c} h_{y}=\left\langle y^{3}\right\rangle /\left\langle y^{2}\right\rangle \Leftrightarrow \left\langle y^{2}\right\rangle =\left\langle y^{3}\right\rangle /\left\langle y^{2}\right\rangle \end{array}$$ (24)

$$\begin{array}{c} \Leftrightarrow {\left|w\right|}^{2}\left\langle x_{w}^{2}\right\rangle =\left|w\right|\left\langle x_{w}^{3}\right\rangle /\left\langle x_{w}^{2}\right\rangle \end{array}$$ (25)

$$\begin{array}{c} \Leftrightarrow \left|w\right|=\left\langle x_{w}^{3}\right\rangle /{\left\langle x_{w}^{2}\right\rangle }^{2} \end{array}$$ (26)

where *x*_*w*_ = (**w**^*T*^**x**)_+_/|**w**| is the rectified projection of the input **x** on the normalized direction **w**/|**w**|. Notably, the norm of the weight vector does not converge to a predefined value, e.g. as in the original Oja rule [12], but has a final value that depends on the input statistics. For arbitrary parameters *p* > 2 and *r* > *p* − 2, the equivalent of Eq 26 reads:

$$\begin{array}{c} \left|w\right|={\left(\frac{\left\langle x_{w}^{p}\right\rangle }{\left\langle x_{w}^{r}\right\rangle \;\left\langle x_{w}^{2}\right\rangle }\right)}^{1/(r-p+2)} \end{array}$$ (27)

Thus, we have a family of learning rules for different *r* and *p* that all have the same qualitative features.

### Simulations

For the single neuron simulations, we generated three input groups of 20 neurons each. The sparse signal had ON states with a duration of 100ms, with interstimulus intervals following an exponential distribution (time scale *τ*_1_ = 1000*ms*), and added independent Gaussian noise to each neuron. The network signal followed an Ornstein-Uhlenbeck process (time scale *τ*_2_ = 200*ms*), and added independent Gaussian noise. The third group of inputs was generated as independent Gaussian noise. All inputs were mean subtracted, $x_{i}=x_{i}^{\prime }-\langle x_{i}^{\prime }\rangle$, where $x_{i}^{\prime }$ is the *i*-th component of the raw input. For Fig 1, the input standard deviations of each group were *σ*_1_ = 1., *σ*_2_ = 1.2, and *σ*_3_ = 2.2, respectively. For Fig 2A and 2B, the sparse signal inputs were subdivided into three groups with different amplitudes, *σ*_11_ = 1.5, *σ*_12_ = 1., *σ*_13_ = 0.7. For Fig 2F and 2G, the sparse signal inputs were subdivided into three groups with different independent noise amplitudes, $\sigma_{11}^{n}=1.5$, $\sigma_{12}^{n}=1.$, $\sigma_{13}^{n}=0.7$.

The homeostatic factor *h*_*y*_ = 〈*y*^2^〉 was estimated as a moving average of *y*^2^ with time scale of *τ*_*h*_ = 200 samples: $h_{t}=h_{t-1}\;\left(1-1/\tau_{h}\right)-y_{t}^{2}/\tau_{h}$. All simulations generated 10^6^ data samples and ran the learning model for 10^6^ time steps. We implemented stochastic gradient descent updates using the Adam optimizer with learning rate *η* = 0.003, mini-batches with 100 random samples, and random initial weights with a Gaussian distribution of mean zero and unit variance.

For the population coding simulations, we generated the latent variable from a random walk, smoothed with an exponential filter (time scale *τ*_3_ = 100*ms*), with circular values, by clipping to [0, 1]. We generated 100 inputs, with evenly spaced Gaussian tuning curves, with 0.05 width, including additive independent Gaussian noise to the input activities (*σ* = 0.01). For generating heterogeneous tuning curves, we scaled the noise, width and amplitude of each tuning curve by independent log-normal random variables, with zero mean and *σ* = 0.2. The population network included 16 output neurons. We included all-to-all inhibitory recurrent connections $w_{ij}^{rec}$ from neuron *j* to neuron *i*, without self-connections. Each neuron had activation $y_{j}={\left(w^{T}x+w_{rec}^{T}y\right)}_{+}$, with inhibitory plasticity $\Delta w_{ij}^{rec}=-\eta^{rec}\left(y_{i}\left(y_{j}-\theta \right)-\lambda^{rec}w_{ij}^{rec}\right)$, clipped to negative values only, with λ^*rec*^ = 1.0, *θ* = 1., *η*^*rec*^ = 0.03. To maintain network stability, we also added weight decay to the feedforward plasticity model, $\Delta w_{t}=\eta \left(x_{t}y_{t}^{2}-h_{y}x_{t}y_{t}-\lambda w\right)$, with λ = 0.001. For each input sample, we ran the recurrent dynamics for 10 time steps.

For the spiking network, we generated 16x16 image patches, sampled from black and white natural images [4], divided into ON and OFF cells, totalling 512 input neurons. Input spike trains were generated as Poisson processes, with the rate modulated by the pixel amplitude, and 100ms duration per data sample. 64 output neurons were simulated as standard leaky integrate-and-fire neurons, with *V*_*rest*_ = −65*mV*, *V*_*threshold*_ = −50, *V*_*reset*_ = −65*mV*, *τ*_*mem*_ = 15*ms*. We simulated an input mean cancellation mechanism through a negative input current with its amplitude following an estimate of the input firing rate, calculated as the moving average of the input spike train with time scale *τ*_4_ = 200*s*. Short-term depression [34] and spiking threshold adaptation [35] are possible mechanisms for an effective mean subtraction in cortical neurons.

The minimal triplet-STDP model [25] was implemented with weight decay and a homeostatic factor, in which synaptic changes follow

$$\begin{array}{c} \frac{d}{dt}w\left(t\right)=\eta^{+}y\left(t\right){\bar{y}}^{+}\left(t\right){\bar{x}}^{+}\left(t\right)-\eta^{-}h_{y}x\left(t\right){\bar{y}}^{-}\left(t\right)-\lambda w\left(t\right) \end{array}$$ (28)

where *y*(*t*) and *x*(*t*) are the post- and pre-synaptic spike trains, respectively: *y*(*t*) = ∑_*f*_*δ*(*t* − *t*^*f*^), where *t*^*f*^ are the firing times and *δ* denotes the Dirac *δ*-function; *x*(*t*) is a vector with components $x_{i}\left(t\right)=\sum_{f}\delta \left(t-t_{i}^{f}\right)$, where $t_{i}^{f}$ are the firing times of pre-synaptic neuron *i*. *η*^+^ = 10^−4^, *η*^−^ = 10^−4^ and λ = 0.05 are unit-free constants, and ${\bar{y}}^{+}$, ${\bar{x}}^{+}$ and ${\bar{y}}^{-}$ are moving averages, implemented by integration (e.g. $\tau \frac{\partial \bar{y}}{\partial t}=-\bar{y}+y$), with time scales of 30 ms. The homeostatic factor *h*_*y*_ = 〈*y*〉^2^, estimated with a time scale *τ*_*h*_ = 200*s*. The variation of the triplet STDP model with heterosynaptic LTD was composed of the triplet LTP factor and a heterosynaptic LTD factor,

$$\begin{array}{c} \frac{d}{dt}w\left(t\right)=\eta^{+}y\left(t\right){\bar{y}}^{+}\left(t\right){\bar{x}}^{+}\left(t\right)-\eta^{-}w\left(t\right)h_{y} \end{array}$$ (29)

with *η*^+^ = 10^−4^, *η*^−^ = 10^−4^ and *h*_*y*_ = 〈*y*〉^2^, estimated with a time scale *τ*_*h*_ = 200*s*.

Recurrent inhibitory plasticity was adapted from [48], with weight decay, with synaptic changes following

$$\begin{array}{c} \frac{d}{dt}w\left(t\right)=\eta \;(\bar{x}\left(t\right)\left(y\left(t\right)-\theta \right)+x\left(t\right)\left(\bar{y}\left(t\right)-\theta \right))-\lambda w\left(t\right) \end{array}$$ (30)

with constants *η* = 0.001, *θ* = 0.003 and λ = 3.0.
